# Round the Hospitals

**Published:** 1918-01-05

**Authors:** 


### ROUND THE HOSPITALS.

THE fourth year of the war will be memorable in history, for personal service was crowned with glory by the united, whole-hearted surrender of themselves on the part of hospital residents, matrons, sisters, and nurses, with others, who resolved that every hospital inmate should forget the war, have a blessed Christmas and subsequent days, simple, heart-stirring and abiding. Forty-nine Christmas days in a hospital rejoice the heart of the writer, because the unity of hospital effort in 1917 produced probably the most joyous Christmas in the lives of hospital patients yet achieved. This splendid outpouring of personal service warms the heart and makes us wish for all a New Year full of blessedness, contentment and hope.

THE Bolos of the nursing world have been working hard to assert themselves and keep on their feet by trying to attract the notice of the public. Their complete failure is demonstrated by two facts: One is, that their case is so weak that they are in the habit of continuously sending for publication the same inaccurate statements in a greatly exaggerated form, or minimised to destruction by the proved falseness of many of the statements, which is demonstrated by earlier correspondents whose communications have been published. When thus exposed to the indignation of the nursing profession and the ridicule of the public, they exhibit their spleen by scurrilous abuse of those who have proved themselves to be the best friends trained nurses have, and of new friends

☛ Previous articles appeared December 8, 15 and 22.

**ROUND THE HOSPITALS—(*continued*).**

who, like Lady Cowdray and the British Women's Hospital Committee, are rendering magnificent personal service with great self-denial and the sole wish and object of raising a Building Fund for the College of Nursing, and a Nurses' Tribute Fund for the protection and strengthening of the position of those members of the nursing profession who may find themselves incapacitated through no fault of their own, and are so entitled to every possible help that money can afford. These Bolos have so outraged public and nursing opinion that any influence they might have possessed is rapidly approaching the vanishing-point. Meanwhile, every trained nurse who loves her profession and honours her friends should rally round the College, as indeed trained nurses are doing in increasing numbers every week, and devote her energies, influence, and time to the utmost extent in making the facts known to her sisters, and so securing that the Register of the College shall contain the names of 15,000, or, better still, 20,000, of the best of nurses within the shortest possible time. To do less than this is for each trained nurse, who remains indifferent, to stand in her own light, to turn her back upon her vital interests, and to do her best to delay State Registration and the granting of full justice to trained nurses everywhere.

---

THE second fact is that a new policy has been started by the Bolos by circulating statements, which the Press are worried to publish, containing either direct falsehoods or suggesting reasons or positions which are false. The latest instance of the latter is a paragraph which appeared in the *Times* on the 27th ultimo headed "The Status of Nurses," which contained the statement that "the Charter" of the Royal British Nurses' Association, "as amended by the Privy Council, made it possible to admit nurses" to the Register "who had not undergone the three years' training which" the Royal British Nurses' Association "considers essential to its members' interests." The character of this contention is nailed to the counter by the Hon. Sir Arthur Stanley, G.B.E., M.P., whose letter under the heading "The Status of Nurses" appeared in the *Times* on the 29th ultimo. Here it is:—

SIR,—My attention has been called to a paragraph in your issue of the 26th inst. in which it is implied that the provisions of the Supplemental Charter which the Royal British Nursing Association had approved as a basis of amalgamation with the College of Nursing were so altered by the Privy Council as no longer to secure the three years' training for nurses "which the Association considers essential to its members' interests." To show how devoid of substance is this contention I venture to quote the relevant clauses as they stand in the Supplemental Charter side by side with the changes proposed by the Privy Council:—

The purposes of the Corporation shall be extended so as to include the following:—

(*b*) The improvement of the training, education, and professional status of nurses and the promotion of a uniform curriculum and standard of qualification.

The Privy Council substitutes:—

. . . and the promotion of equivalent curricula and standards of qualification for all classes of nurses.

(*d*) The making and maintaining of an official register of persons qualified to act as nurses.

The Privy Council substitutes:—

The making and maintaining of a register of persons qualified, in the opinion of the corporation, to act as nurses.

These parallel quotations place your readers in a position to judge for themselves how far the action of the Privy Council diminished the safeguards as to training which satisfied the Royal British Nursing Association a year ago. I fear that the true reasons for repudiating their agreement to amalgamate with the College of Nursing must be sought elsewhere.—Yours, etc., ARTHUR STANLEY, Chairman, Council of College of Nursing.

---

THE Bolos' action and methods demonstrate the contempt in which they hold the intelligence of trained nurses in this country. Is it not time that trained nurses aroused themselves and marshalled their forces by unity and action to defeat the Bolos? The College Register is open to every trained nurse. Sir Arthur Stanley, who possesses the confidence of the House of Commons, the respect and gratitude of the Nation and Empire, and the unanimous support of those who have given trained nursing of the best to the people, is a friend and leader they may rejoice to follow with absolute confidence, with the certainty of victory for the causes which they have properly so much at heart. The College Register is the Register open to the trained nurse, which rests upon the highest standards and has been set up by her trusted teachers, leaders, and friends, with the supreme object of helping trained nurses to help themselves, and afford them and their profession the strength, with the advantages, so far as they are interchangeable, which is already possessed by the members of the medical profession. If trained nurses in this country have the grit which mere men believe them to possess, 20,000 nurses at least will register with the College of Nursing without a moment's longer hesitation or delay. For any trained nurse to do less than this is to demonstrate to Parliament that nurses either do not desire to have State Registration, or that the majority are unfit to warrant Parliament in passing an Act in existing circumstances.

---

**☞ IT is our invariable practice to pay for all items of news which we may publish. The minimum payment is 5s. Paragraphs of about twenty lines in length—that is to say, of 150 words or less—are preferred. Contributions should be addressed to The Editor, THE HOSPITAL, 28 and 29 Southampton Street, Strand, London, W.C. 2, and, to be in time for the current week's issue, should reach him by the first post on Mondays.**

